# Selective internal radiotherapy and chemosaturation show equivalent survival in metastatic uveal melanoma: a retrospective multicenter study

**DOI:** 10.1093/oncolo/oyag152

**Published:** 2026-05-08

**Authors:** Elias A T Koch, Louisa A Schielein, Markus Reitmajer, Ann-Christine Glaser, Konstantin Drexler, Cindy Franklin, Andrea Forschner, Carola Berking, Jessica C Hassel, Markus V Heppt, German Dermatologic Cooperative Oncology Group (DeCOG, Committee Ocular Melanoma), Armin Bender, Christoffer Gebhardt, Martin Gschnell, Lucie Heinzerling, Nicole Kreuzberg, Max Schlaak, Dirk Tomsitz

**Affiliations:** Department of Dermatology, Uniklinikum Erlangen, Friedrich-Alexander-University Erlangen-Nürnberg (FAU), Erlangen, 91054, Germany; Comprehensive Cancer Center Erlangen—European Metropolitan Area of Nuremberg (CCC ER-EMN), Erlangen, 91054, Germany; Deutsches Zentrum Immuntherapie (DZI), Uniklinikum Erlangen, Friedrich-Alexander-University Erlangen-Nürnberg (FAU), Erlangen, 91054, Germany; Bavarian Cancer Research Center (BZKF), Uniklinikum Erlangen, Östliche Stadtmauerstraße 30, Erlangen, 91054, Germany; Heidelberg University, Medical Faculty Heidelberg, Department of Dermatology and National Center for Tumor Diseases (NCT), NCT Heidelberg, a partnership between DKFZ and University Hospital Heidelberg, Heidelberg, 69120, Germany; Department of Dermatology, Center for Dermatooncology, University Hospital Tübingen, Tübingen, 72076, Germany; Department of Dermatology and Venereology, University of Cologne, Faculty of Medicine and University Hospital Cologne, Cologne, 50937, Germany; Department of Dermatology, University Hospital Regensburg, Regensburg, Germany; Department of Dermatology and Venereology, University of Cologne, Faculty of Medicine and University Hospital Cologne, Cologne, 50937, Germany; Department of Dermatology, Center for Dermatooncology, University Hospital Tübingen, Tübingen, 72076, Germany; Department of Dermatology, Uniklinikum Erlangen, Friedrich-Alexander-University Erlangen-Nürnberg (FAU), Erlangen, 91054, Germany; Comprehensive Cancer Center Erlangen—European Metropolitan Area of Nuremberg (CCC ER-EMN), Erlangen, 91054, Germany; Deutsches Zentrum Immuntherapie (DZI), Uniklinikum Erlangen, Friedrich-Alexander-University Erlangen-Nürnberg (FAU), Erlangen, 91054, Germany; Bavarian Cancer Research Center (BZKF), Uniklinikum Erlangen, Östliche Stadtmauerstraße 30, Erlangen, 91054, Germany; Heidelberg University, Medical Faculty Heidelberg, Department of Dermatology and National Center for Tumor Diseases (NCT), NCT Heidelberg, a partnership between DKFZ and University Hospital Heidelberg, Heidelberg, 69120, Germany; Department of Dermatology, Uniklinikum Erlangen, Friedrich-Alexander-University Erlangen-Nürnberg (FAU), Erlangen, 91054, Germany; Comprehensive Cancer Center Erlangen—European Metropolitan Area of Nuremberg (CCC ER-EMN), Erlangen, 91054, Germany; Deutsches Zentrum Immuntherapie (DZI), Uniklinikum Erlangen, Friedrich-Alexander-University Erlangen-Nürnberg (FAU), Erlangen, 91054, Germany; Bavarian Cancer Research Center (BZKF), Uniklinikum Erlangen, Östliche Stadtmauerstraße 30, Erlangen, 91054, Germany; Dermpath München, Laboratory for Dermatopathology, Oral Pathology and Molecular Pathology, Munich, 80335, Germany

**Keywords:** uveal melanoma, metastatic uveal melanoma, liver-directed therapies, selective internal radiotherapy, percutaneous hepatic perfusion, real-world evidence, multidisciplinary tumor board

## Abstract

**Background:**

Metastatic uveal melanoma (UM) primarily affects the liver, and due to low response rates to systemic therapies, liver-directed therapies (LDT) are commonly used for hepatic tumor control. Direct head-to-head trials comparing these LDTs are still lacking.

**Methods:**

A retrospective multicenter explorative analysis was conducted to evaluate the clinical outcomes of transarterial chemoembolization (TACE), selective internal radiotherapy (SIRT), and percutaneous hepatic perfusion (PHP, chemosaturation).

**Results:**

The present analysis was conducted on a cohort of 121 patients who had been treated for metastatic UM with LDT at five different skin cancer centers. A total of *n* = 15 patients received TACE (12.4%), *n* = 51 SIRT (42.1%), and *n* = 55 PHP (45.5%). The estimated median overall survival (OS) for SIRT was 25.1 months (95% confidence interval [CI], 15.3-34.8), for TACE, 11.4 months (95% CI, 7.8-14.9), and for PHP, 24.9 months (95% CI, 13.7-36.0). In the multivariate analysis that included LDH and additional systemic therapies, there was no significant difference in OS between SIRT and PHP (HR 1.14, 95% CI 0.67-1.93, *P* = .63), whereas TACE was found to be associated with a substantially reduced in OS compared to PHP (HR 3.25, 95% CI 1.31-8.04, *P* = .01). Adverse events occurred in 41.6% of patients undergoing SIRT, 64.3% of patients receiving TACE, and 84.6% of patients receiving PHP.

**Conclusion:**

The findings of this study indicate that SIRT and PHP demonstrate equivalent survival rates in a real-world setting.

Implications for Practice In patients with metastatic uveal melanoma and liver-dominant disease, liver-directed therapies are frequently applied. In the absence of prospective head-to-head trials, this multicenter real-world analysis provides comparative outcome data on commonly used liver-directed options, which may aid multidisciplinary tumor boards in selecting and integrating these therapies into multimodality treatment strategies. In this context, selective internal radiotherapy and percutaneous hepatic perfusion may be preferentially considered, depending on institutional expertise and available resources.

## Introduction

Uveal melanoma (UM) represents the most common malignant tumor of the eye in adults, yet it remains a rare condition, with an incidence of about five cases per million in Caucasian populations.^1^ Genetic alterations play a decisive role in disease course, and nearly half of all patients develop metastatic spread.^2^^,^^3^ Once metastases appear, survival outcomes decline sharply.^4^^,^^5^ Immune checkpoint inhibitors (ICB), such as antibodies targeting PD-1 or CTLA-4, have provided encouraging results in retrospective studies in patients with metastatic UM, while tebentafusp has been the first drug to show a clear overall survival (OS) benefit in a prospective, controlled, randomized setting.^6^^,^^7^ The pivotal trial revealed a median OS of 21.6 months compared with 16.9 months in the control group (HR 0.68; 95% CI, 0.54-0.87).^8^^,^^9^ Despite this improvement, the prognosis remains poor, not least because tebentafusp is restricted to individuals carrying the HLA-A*02:01 allele.^9^ In more than 90% of patients with UM, metastases primarily occur in the liver, with liver progression being the key factor limiting survival.^7^ Liver metastases and liver-dominant disease represent the metastatic site with the poorest response to immunotherapy, in clear contrast to extrahepatic metastases, which generally demonstrate a more favorable therapeutic effect.^10^ This unique biology has led to the widespread use of liver-targeted approaches in metastatic UM.^11^ The most common procedures are currently transarterial chemoembolization (TACE), selective internal radiotherapy (SIRT), and percutaneous hepatic perfusion (PHP, also known as chemosaturation). TACE is frequently employed for larger solitary lesions, but the absence of comparative clinical trials and the lack of standardized treatment protocols continue to pose significant challenges for its optimal application and selection.^12^ In cases of disseminated liver metastases, as well as depending on the distribution pattern and location-specific availability of therapeutic options, SIRT, and PHP are typically employed. SIRT and PHP are both liver-directed therapies (LDT) that have shown promising outcomes in clinical studies. SIRT demonstrated median OS of up to 18.5 months, while PHP with melphalan has achieved a median OS of 20.5 months in prospective trials, particularly after safety improvements with the second generation (GEN2) filter.^13^^,^^14^ However, as direct head-to-head trials comparing these LDT modalities are still lacking, we addressed this knowledge gap by conducting a retrospective analysis in order to evaluate their comparative clinical outcomes.

## Methods

### Patient population and study design

We performed a retrospective multicenter explorative analysis. Patients with metastatic UM receiving LDT (SIRT, TACE, or PHP) were eligible. Clinical data and the treatment outcomes of interest were extracted from original patient records at five German skin-cancer centers (Erlangen *n *= 48, Heidelberg *n *= 35, Tübingen *n *= 25, Köln *n *= 10, Regensburg *n *= 3). Apart from the presence of hepatic metastases and the administration of at least one LDT modality, no additional eligibility criteria were applied. The study cohort included patients treated between April 2009 and July 2025. The selection of a certain type of LDT was left to the discretion of each participating center and was determined by a multidisciplinary tumor board. If a patient received more than one type of LDT during the course of the disease, analyses were based on the first LDT applied. The data were collected, checked for duplicates, and merged into a central database before analysis. This study was approved by the institutional review board of the medical faculty of the Munich University Hospital (approval number 413-16 UE), and it was conducted following the principles of the Helsinki Declaration in its current version.

### Data collection and treatment outcomes

Baseline clinical data included demographic variables (sex, age), the number of organ systems involved by metastatic disease, and the date of death or last documented patient contact. At initiation of LDT, serum lactate dehydrogenase (LDH) levels were extracted from patient records and evaluated for their prognostic relevance. LDH was measured locally at each participating center and recorded in U/L. One center used sex-specific reference ranges, with an upper limit of normal of <301 U/L for women and <317 U/L for men, whereas the other centers used an upper limit of normal of approximately 250 U/L. Treatment-related information comprised the number and type of administered therapies, the date of LDT administration, the best radiologic response to LDT assessed according to RECIST version 1.1 based on contrast-enhanced CT imaging and/or MRI as documented in the respective clinical records. Complete remission (CR), partial remission (PR), and stable disease (SD) were summarized as disease control rate (DCR), CR and PR as objective response rate (ORR). LDT-related adverse events were documented retrospectively and graded in reference to CTCAE version 5. Metastatic sites other than liver, bone, lung, central nervous system, lymph nodes, connective tissue, and skin were summarized under the category “other metastases.”

### Statistical analyses

Time-to-event analyses were conducted, with death considered as event of interest. OS was defined as the interval from initiation of the first applied LDT to death from any cause. Patients who were alive at last follow-up or lost to follow-up were censored at the date of their last documented contact. Survival probabilities were estimated using the Kaplan–Meier method and Cox regression analysis. Univariate group differences in survival and progression were assessed with log-rank tests. In the Cox regression models, hazard ratios (HR) with 95% confidence intervals (CI) were calculated, and statistical significance of individual predictors was assessed using Wald tests. For Cox regression, LDTs were entered as the main variable, with PHP, as the largest group, defined as the reference category. Additional therapies (tebentafusp, chemotherapy, dendritic cell vaccine, radiation therapy, ICB, and other systemic therapies) as well as LDH were included as covariates to adjust for potential confounding. Furthermore, Pearson’s Chi-square tests were conducted to assess group differences in categorical variables. If the assumptions of the Chi-square test were violated (eg, low expected cell frequencies), the Fisher–Freeman–Halton exact test was applied instead. Group differences in continuous variables were analyzed using one-way ANOVA. *P*-Values were reported from the global ANOVA and, where applicable, after Bonferroni adjustment for multiple comparisons. Ordinal variables were compared across the three treatment arms using the nonparametric Kruskal–Wallis test. All tests were two-sided, and *P*-values <.05 were considered statistically significant. Statistical analyses and graphical representations were performed using IBM SPSS Statistics version 28.0 (IBM Corp., Armonk, NY, USA) and R version 4.4.3 with the survival, survminer, and forestmodel packages.

## Results

### Baseline patient characteristics

A total of 121 patients with metastatic UM undergoing LDT were included, of whom *n* = 15 (12.4%) received TACE, *n* = 51 (42.1%) SIRT, and *n* = 55 (45.5%) PHP. The serum LDH was elevated in 58.7% of cases (*n* = 71) at baseline. All patients presented with liver metastases (100%). Additional metastatic sites included the lung in 24.8% of patients, bone in 20.7%, lymph nodes in 15.7%, connective tissue in 13.2%, skin in 6.6%, and the central nervous system in 1.7%. Metastases at other sites were observed in 19% of patients. The patient group treated with TACE showed significant heterogeneity in the protocols used, both in terms of active substances and the embolization method. Regarding the technique, nine patients underwent bilobar TACE, seven were treated using degradable starch microspheres (DSM-TACE), and one patient received a segmental TACE. The treatments were performed with melphalan in six, cisplatin in two, irinotecan in one, and a combination of mitomycin, doxorubicin, and embocept in four patients.

Pairwise comparisons showed that patients in the SIRT group were significantly older than in the PHP group (*P* = .007) and comprised a markedly higher proportion of patients with liver-only metastases (*P* < .001). Notably, no significant difference in LDH values was observed between the two groups (*P* = .633). Patients in the TACE group were older compared to those in the PHP (*P* < .001) and SIRT groups (*P* = .068). The TACE group also presented with a higher proportion of extrahepatic metastases compared with the SIRT group (*P* < 0.001), whereas the difference to the PHP group was not significant (*P* = .121). Baseline LDH levels were lower in the TACE group, but did not differ significantly from the PHP group (*P* = .141) or the SIRT group (*P* = .718). All baseline characteristics are listed in detail in Table 1.

**Table 1.** Baseline characteristics of the study population.

	SIRT (*n* = 51)		**TACE (*n* = 15)**		PHP (*n* = 55)	
	Missing		Missing		Missing	
**Age (years), mean ± std. dev.**	–	65.2 ± 10.9	–	72.5 ± 9.9	–	58.6 ± 10.9
**Sex, *n* (%)**	0 (0%)	–	0 (0%)	–	0 (0%)	–
** Female**	–	34 (66.7%)	–	9 (60%)	–	31 (56.4%)
** Male**	–	17 (33.3%)	–	6 (40%)	–	24 (43.6%)
**Metastases, *n* (%)**						
** Liver**	0 (0%)	51 (100%)	0 (0%)	15 (100%)	0 (0%)	55 (100%)
** Pulmonary**	0 (0%)	9 (17.6%)	0 (0%)	3 (20%)	0 (0%)	18 (32.7%)
** Bone**	0 (0%)	2 (3.9%)	0 (0%)	1 (6.7%)	0 (0%)	22 (40%)
** CNS**	0 (0%)	0 (0%)	0 (0%)	0 (0%)	0 (0%)	2 (3.6%)
** Lymph node**	0 (0%)	3 (5.9%)	0 (0%)	0 (0%)	0 (0%)	16 (29.1%)
** Soft tissue**	0 (0%)	2 (3.9%)	0 (0%)	1 (6.7%)	0 (0%)	13 (23.6%)
** Skin**	0 (0%)	1 (2%)	0 (0%)	1 (6.7%)	0 (0%)	6 (10.9%)
** Other location**	3 (5.9%)	2 (3.9%)	0 (0%)	0 (0%)	0 (0%)	1 (1.8%)
**Extrahepatic metastases, *n* (%)**	0 (0%)	11 (21.6%)	0 (0%)	13 (86.7%)	0 (0%)	35 (63.6%)
**Liver-only metastases, *n* (%)**	0 (0%)	40 (78.4%)	0 (0%)	2 (13.3%)	0 (0%)	20 (36.4%)
**LDH (elevated), *n* (%)**	0 (0%)	33 (64.7%)	1	3 (20%)	2 (3.6%)	35 (63.6%)
**LDH value, mean ± std. dev.**	–	417.6 ± 418.2	–	262.8 ± 101.8	–	526.4 ± 500.8
**Cycles of LDT, *n* (%)**	0 (0%)	–	0 (0%)	–	0 (0%)	–
** 1**	–	24 (47.1%)	–	6 (40%)	–	15 (27.3%)
** 2**	–	24 (47.1%)	–	4 (26.7%)	–	27 (49.1%)
** 3**	–	2 (3.9%)	–	3 (20%)	–	7 (12.7%)
** 4**	–	1 (2%)	–	0 (0%)	–	6 (10.9%)
** 5**	–	0 (0%)	–	2 (13.3%)		0 (0%)

Abbreviations: CNS = central nervous system, LDT = liver-directed therapies, PHP = percutaneous hepatic perfusion (also chemosaturation), SIRT = selective internal radiotherapy, TACE = transarterial chemoembolization.

Most patients received only one liver-directed treatment modality, reflecting that treatment was generally delivered within a single center with expertise predominantly focused on one specific procedure. However, a subset of patients underwent additional LDT at different time points after the initial treatment. A detailed overview of these subsequent liver-directed procedures is provided in Table 2.

**Table 2.** Subsequent liver-directed treatments according to the initial treatment applied (PHP, SIRT, and TACE). Other LDTs mainly comprised radiofrequency ablation and liver segment resection.

			Liver-directed therapy		
			SIRT	TACE	PHP
**Additional LDT**	None	Count	42	11	47
		% within LDT	82.4%	73.3%	85.5%
	SIRT	Count	–	0	2
		% within LDT	–	0%	3.6%
	TACE	Count	3	–	1
		% within LDT	5.9%	–	1.8%
	PHP	Count	0	3	–
		% within LDT	0%	20%	–
	Other LDT	Count	6	1	5
		% within LDT	11.8%	6.7%	9.1%
**Total**		Count	51	15	55
		% within LDT	100%	100%	100%

Abbreviations: PHP = percutaneous hepatic perfusion (also chemosaturation), SIRT = selective internal radiotherapy, TACE = transarterial chemoembolization; LDT = liver-directed treatment.

### Response rates

The PHP group achieved the most favorable outcomes, with a DCR of 74.9% (26.8% PR, 48.1% SD), while the TACE group reached a DCR of 50.0% (42.9% PR, 7.1% SD). In contrast, the SIRT group showed the lowest response, with a DCR of 32% (22% PR, 10% SD). Progressive disease (PD) was more common following SIRT (52%) and TACE (42.9%), but notably lower with PHP (25%). The distribution of RECIST response categories differed significantly between the groups (Fisher–Freeman–Halton exact test, *P* < .001). Further information about the response rates to LDTs is listed in Table 3.

**Table 3.** Response rates to LDT.

	SIRT (*n* = 50/51; 1 missing)	TACE (*n* = 14/15; 1 missing)	PHP (*n* = 52/55; 3 missing)
**Best response**	*n* (%)	*n* (%)	*n* (%)
** CR**	0 (0%)	0 (0%)	0 (0%)
** PR**	11 (22%)	6 (42.9%)	14 (26.8%)
** SD**	5 (10%)	1 (7.1%)	25 (48.1%)
** PD**	26 (52%)	6 (42.9%)	13 (25%)
** MR**	8 (16%)	1 (7.1%)	0 (0%)
** ORR**	11 (22%)	6 (42.9%)	14 (26.8%)
** DCR**	16 (32%)	7 (50%)	39 (74.9%)

Abbreviations: CR = complete response, DCR = disease control rate, MR = mixed response, ORR = objective response rate, PD = progressive disease, PHP = percutaneous hepatic perfusion (also chemosaturation), PR = partial response, SD = stable disease, SIRT = selective internal radiotherapy, TACE = transarterial chemoembolization.

### Survival data

**Figure 1.**
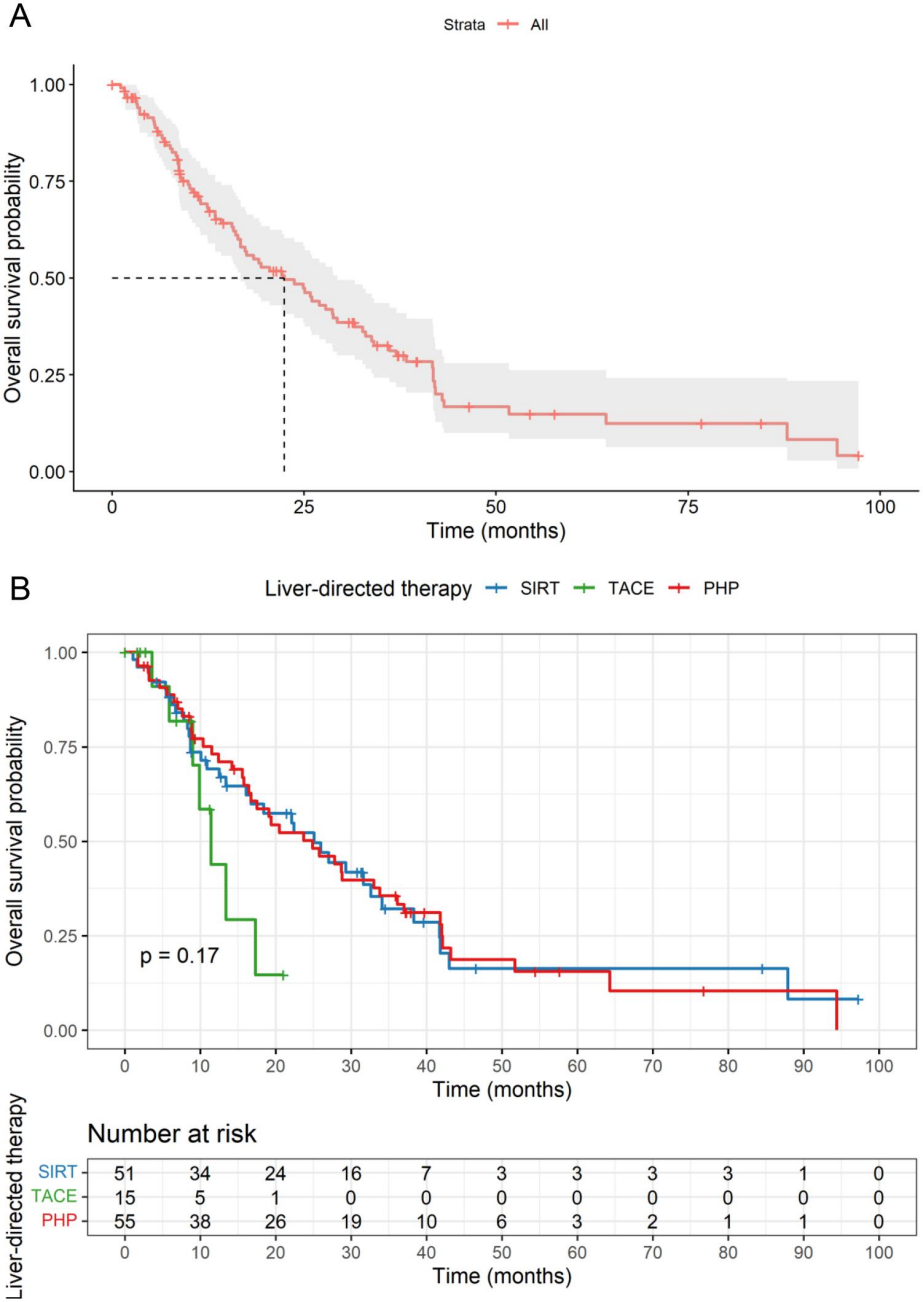
(A) Kaplan–Meier curve for overall survival in the entire cohort. Estimated median overall survival: 22.4 months (95% CI 15.5-29.2). (B) Kaplan–Meier curves for overall survival stratified by the LDT groups. Survival probabilities were calculated unadjusted, with differences between groups reflecting observed events and censoring. Estimated median overall survival: for SIRT 25.1 months (95% CI 15.3-34.8); for TACE: 11.4 months (95% CI 7.8-14.9); for PHP: 24.9 months (95% CI 13.7-36). Number at risk at selected time points for each treatment arm.

**Figure 2.**
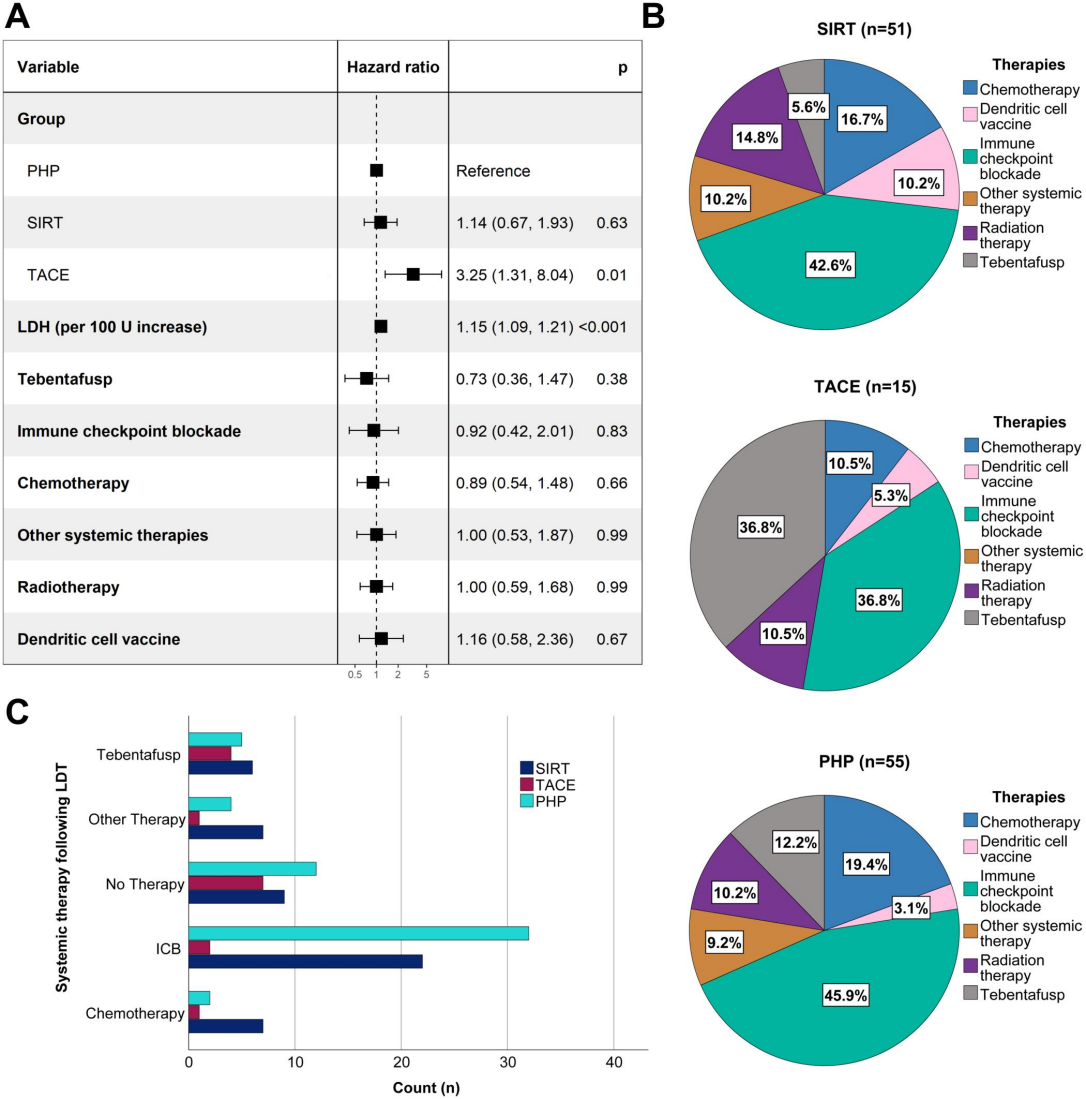
(A) Forest plot of Cox proportional hazards regression analysis. Hazard ratios with 95% confidence intervals are shown for each covariate. Among all covariates tested, only LDH (per 100-unit increase) showed statistically significant results. (B) Distribution of systemic therapies received by patients in the LDT groups throughout the course of the disease, regardless of whether before or after LDT. Pairwise comparisons showed that there were no significant differences between the SIRT and PHP groups regarding the administration of tebentafusp (*P* = .168), ICB (*P* = .216), chemotherapy (*P* = .936), other systemic therapy (*P* = .494), or radiation therapy (*P* = .115). However, in the SIRT group, 10.2% of patients received dendritic cell vaccination, which was significantly more frequent compared to the PHP group (*P* = .014). (C) Grouped bar plot of subsequent systemic therapies administered after the first liver-directed treatment, shown separately for the PHP, SIRT, and TACE treatment arms. Bars represent the number of patients in each category.

The entire cohort showed an estimated median OS of 22.4 months (95% CI 15.5-29.2; Figure 1A). There was no statistical difference in OS between all groups (*P* = .17; Figure 1B), but a strong trend for poorer survival in the TACE group. In the multivariable Cox regression analysis, no difference in OS was observed in comparison to SIRT (HR 1.14, 95% CI 0.67-1.93, *P* = .63), whereas TACE was associated with a significantly lower OS (HR 3.25, 95% CI 1.31-8.04, *P* = .01). Higher LDH at the time of LDT was associated with lower OS (per 100-unit increase: HR 1.15, 95% CI 1.09-1.21, *P* < .001). Other covariates were not significantly related to OS. All point estimates and 95% confidence intervals are shown in the forest plot in Figure 2A.

To further assess the impact of extrahepatic metastases as potential confounder, we performed an additional multivariable Cox regression analysis including LDH and the presence of extrahepatic metastases. In this model, the hazard ratio for SIRT compared with PHP was 1.02 (95% CI 0.61-1.73), indicating no significant difference in OS after adjustment for these covariates (Supplementary Figure S1).

### Adverse events

Adverse events (AE) were reported in 73 patients (60.3%) and they were estimated as severe in 23 patients (19%). The incidence of AE differed significantly between the groups (Pearson’s Chi-square test, χ^2^(2) = 19.99, *P* < .001). For further information see Table 4.

**Table 4.** Occurrence of adverse events (AE).

	SIRT (*n* = 48/51; 3 missing)	TACE (*n* = 14/15; 1 missing)	PHP (*n* = 52/55; 3 missing)
**Adverse events (*n*)**	20 (41.6%)	9 (64.3%)	44 (84.6%)
**CTCAE Grade (*n*)**			
** 1**	17 (35.4%)	2 (14.3%)	9 (17.3%)
** 2**	4 (8.3%)	6 (42.9%)	14 (26.9%)
** 3**	1 (2.1%)	1 (7.1%)	12 (23.1%)
** 4**	0 (0%)	0 (0%)	9 (17.3%)

Abbreviations: CTCAE = Common Terminology Criteria for Adverse Events, PHP = percutaneous hepatic perfusion (also chemosaturation), SIRT = selective internal radiotherapy, TACE = transarterial chemoembolization.

## Discussion

SIRT has been evaluated as a treatment for hepatic metastases of UM, with initially a retrospective study of *n* = 71 patients showing a median OS of 12.3 months and a progression-free survival (PFS) of 5.9 months.^15^ A subsequent prospective phase II trial reported markedly improved outcomes, with a median OS of 18.5 months and a PFS of 8.1 months.^14^^,^^15^ Further, PHP revealed improved hepatic responses in early trials compared to the best alternative care. However, the initial survival benefit was limited, and treatment-related mortality occurred.^16^ With the improved GEN2 filter, safety increased, and in a phase II trial with liver-only metastases, a median OS of 19.1 months and PFS of 7.6 months were achieved.^13^^,^^17^ The following phase III FOCUS trial demonstrated a significant advantage of PHP over best alternative care, with a median OS of 20.5 months versus 14.1 months and a median PFS of 9.0 months versus 3.1 months, respectively.^18^^,^^19^ To our knowledge, there is only one retrospective single-center study comparing PHP with SIRT.^20^ The study showed an improved median OS of 17.2 months for PHP compared to SIRT with a median overall survival of 10 months (*P* = .006).^20^ In addition, the median PFS was 4.25 months for SIRT and 13.6 months for PHP (*P* = .090).^20^ In comparison, our analysis showed no significant difference in OS between SIRT and PHP, even when taking several covariates into account. Notably, the SIRT and PHP groups were comparable with respect to baseline LDH levels, as LDH represented the strongest independent factor for OS in the multivariate analysis. Further, several interventional oncology procedures, including SIRT and PHP, have been shown to depend strongly on the expertise of the operator and the center. Evidence suggests that technical execution and patient outcomes are significantly influenced by learning curves and degrees of specialization. For PHP, a clear learning curve was demonstrated, showing that radiation exposure and fluoroscopy times decreased as operators gained experience.^21^ This highlights the importance of consistent performance by the same radiologist to ensure procedural safety and efficiency. Similarly, it was emphasized that PHP is technically demanding and associated with considerable risks.^22^^,^^23^ It was recommended that PHP be performed exclusively in specialized centers with multidisciplinary expertise. Similar findings have been reported for SIRT. Improving patient selection and refining technical protocols and dosimetry has increased the median OS after SIRT for patients with hepatocellular carcinoma from 11.2 to 25.7 months.^24^ Taken together, these studies and the comparable OS rates in this analysis suggest that the efficacy and safety of SIRT and PHP are closely tied to operator and institutional experience. Therefore, the “better” procedure is likely the one with which a given center has more experience. Further, since SIRT and PHP showed similar survival rates, patients undergoing TACE experienced a rapid decline in survival outcomes. In a systemic review, seventeen studies comprising a total of 647 patients evaluated TACE for hepatic metastases from UM, reporting median OS ranging from 5 to 29 months.^12^ Considerable heterogeneity existed among the studies regarding chemotherapeutic agents, techniques, treatment frequency, and the use of additional therapies. This heterogeneity is also reflected in the procedures of our cohort, making direct comparison of outcomes challenging.

Further, our analysis revealed considerable differences in response rates. PD was observed in only 25% of patients following PHP, whereas it was observed in half of the SIRT cases. This observation may be due to the fact that the entire liver is treated under PHP, whereas with SIRT or TACE, only certain segments or lobes of the liver are treated. Further, the response rates to SIRT are lower than those reported in a prospective phase II trial with a DCR of 58.3%.^14^ In contrast, the DCR for PHP in our analysis (74.9%) was consistent with that reported in the phase III trial (73.6%).^19^ However, response rates have been shown to be ineffective as a standalone predictor of survival outcomes since they do not necessarily translate into a meaningful survival benefit.^25^^,^^26^ The discrepancy between OS and ORR is especially apparent in the context of TACE treatment, with an ORR of 42.9% and a comparable low OS. Several factors may explain this discrepancy. TACE is inherently a locoregional treatment that targets only selectively catheterized tumor areas rather than the entire liver or all lesions simultaneously. Consequently, radiologic response in treated lesions may not reflect complete intrahepatic disease control and may have only limited impact on OS. In addition, response rates after TACE may appear comparatively high when assessed using enhancement-based criteria such as mRECIST, which predominantly capture devascularization rather than true anatomic tumor shrinkage. This may further accentuate the dissociation between ORR and OS. Notably, the dissociation between ORR and OS in TACE has also been described in hepatocellular carcinoma (HCC). In a systematic review of 101 studies including 10,108 patients with unresectable HCC treated with conventional TACE, a pooled ORR of 52.5% was reported, whereas median OS was only 19.4 months.^27^ Further, the survival benefit of TACE in HCC itself is not entirely uncontested. In a comparative meta-analysis of 9 randomized trials, no significant survival advantage in the subgroup of studies with low risk of bias was found.^28^ Although these data are not directly transferable to metastatic UM in this cohort, they conceptually support our observation that a decent radiologic response after LDT does not necessarily translate into improved OS.

Moreover, our study revealed that AE occurred in 41.6% of patients undergoing SIRT, 64.3% of patients receiving TACE, and 84.6% of patients receiving PHP. It is noteworthy that only one patient treated with SIRT experienced a severe AE, whereas severe AE occurred in 21 patients (38.2%) treated with PHP, thus emphasizing a considerably higher complication rate in the latter group. The high rate of severe AE in the PHP group is consistent with the results observed in the FOCUS phase 3 trial, where 42.6% of patients experienced treatment-emergent severe AEs.^18^ By contrast, in the prospective phase II trial of SIRT, grade 3 AEs occurred in only 9.8% of cases.^14^ However, due to the retrospective design, AEs may have been underreported in our analysis. Nevertheless, the tendency towards fewer severe AEs under SIRT treatment remains obvious.

The major limitation of this study is its retrospective design and addressing the impact of confounding factors on the prognosis. Although LDH demonstrated a strong prognostic impact and was adjusted for in the multivariate Cox regression, bias and residual confounding cannot be fully excluded, as additional prognostically relevant variables could not be incorporated due to missing data. These variables include ECOG performance status, hepatic tumor burden including assessment of the M stage of the TNM classification, and the size of liver metastases, which may only be partially captured by elevated LDH levels. As previously discussed, operator and center expertise are strongly correlated with outcome, and thus a bias between centers cannot be excluded. Another important limitation is that PFS could not be assessed robustly in this retrospective cohort because progression was not documented in a sufficiently standardized manner across LDT modalities, particularly in the presence of extrahepatic metastatic disease. Furthermore, the limited number of patients receiving TACE, along with the heterogeneity of the procedures in terms of chemotherapeutic agents and technical approaches, represents an important limitation and restricts the ability to draw definitive conclusions from this subgroup, particularly with regard to OS. However, the multicenter real-world design of this study should be considered when interpreting the findings, as it reflects the use of different locoregional treatment strategies for the same condition in the absence of robust comparative evidence. Treatment decisions were not only influenced by clinical factors, but also by local expertise, and the availability of treatment options.

## Conclusion

The findings of the present study demonstrate the first multicenter comparison of SIRT and PHP in a comparatively large cohort. Both were associated with prolonged OS and did not differ between the two groups.

## Supplementary Material

oyag152_Supplementary_Data

## Data Availability

All data generated or analyzed during this study are included in this published article.
