# Supplementary material for: Selective internal radiotherapy and chemosaturation show equivalent survival in metastatic uveal melanoma: a retrospective multicenter study
Source: Oncologist. 2026 May 8;31(6):oyag152. doi: 10.1093/oncolo/oyag152 (PMC13198371; doi:10.1093/oncolo/oyag152)
Supplement: oyag152_Supplementary_Data [file oyag152_supplementary_data.zip › Supplementary figure.docx]

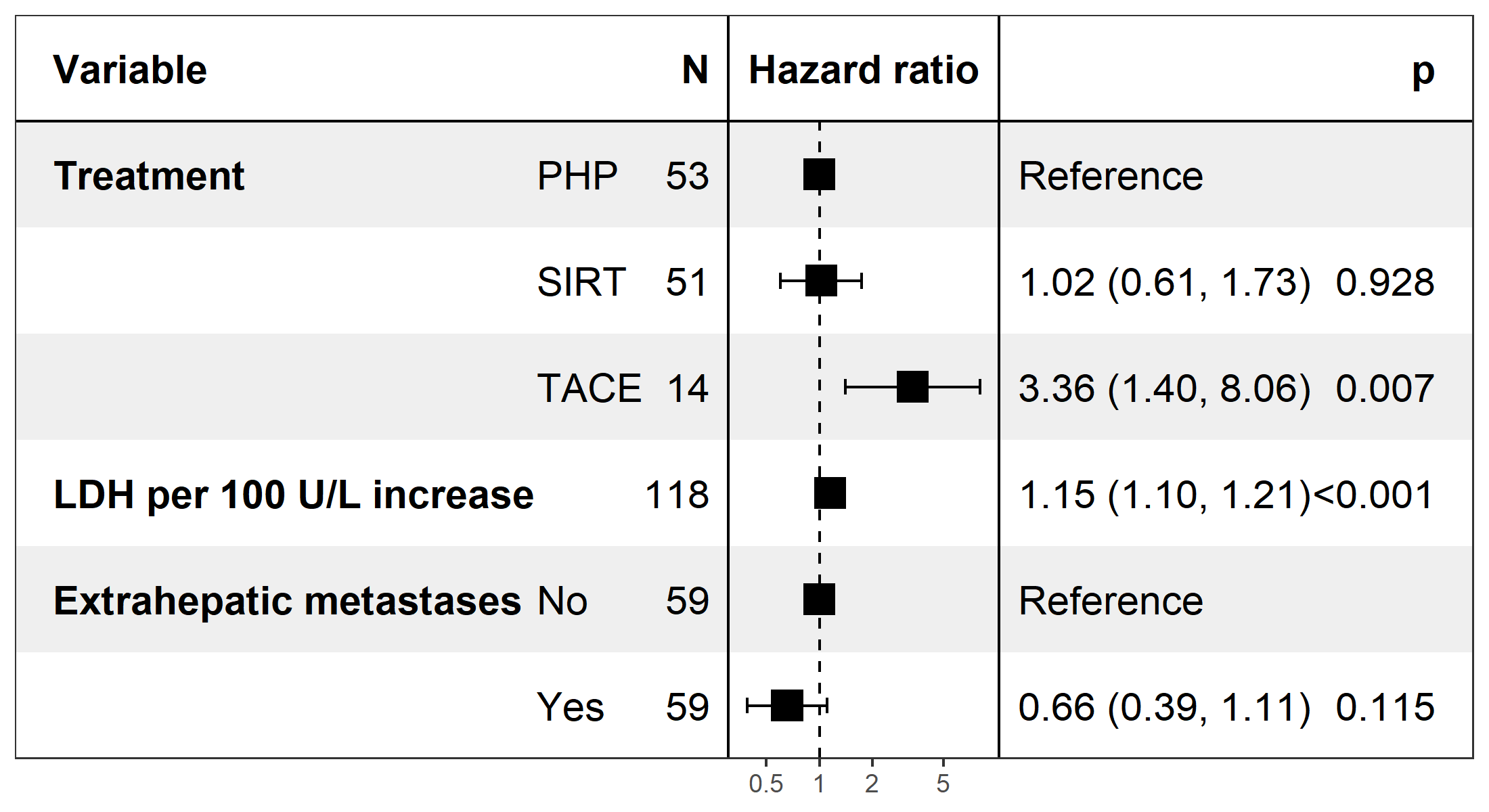


**Supplementary Figure 1. Multivariable Cox regression analysis of overall survival.** Forest plot of the multivariable Cox regression model including treatment arm, LDH, and the presence of extrahepatic metastases. In this model, the hazard ratio for SIRT compared with PHP was 1.02 (95% CI 0.61–1.73), indicating no significant difference in overall survival after adjustment for these covariates.
