# Supplementary figures and images for: Selective internal radiotherapy and chemosaturation show equivalent survival in metastatic uveal melanoma: a retrospective multicenter study
Source: Oncologist. 2026 May 8;31(6):oyag152. doi: 10.1093/oncolo/oyag152 (PMC13198371; doi:10.1093/oncolo/oyag152)

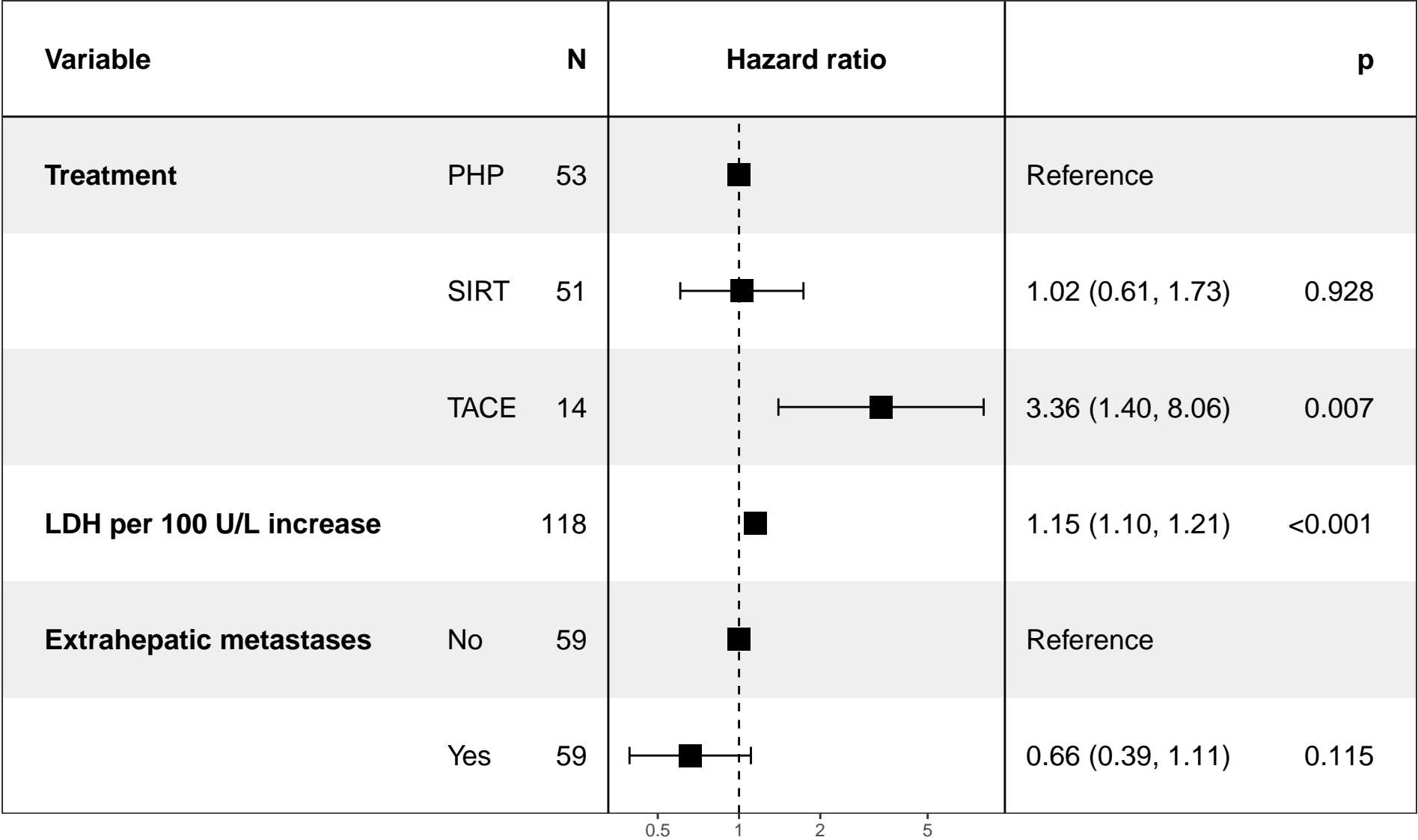

Supplement: oyag152_Supplementary_Data [file oyag152_supplementary_data.zip › Supplementary figure 1.pdf]
